# D-Serine and Glycine Differentially Control Neurotransmission during Visual Cortex Critical Period

**DOI:** 10.1371/journal.pone.0151233

**Published:** 2016-03-22

**Authors:** Claire N. J. Meunier, Glenn Dallérac, Nicolas Le Roux, Silvia Sacchi, Grégoire Levasseur, Muriel Amar, Loredano Pollegioni, Jean-Pierre Mothet, Philippe Fossier

**Affiliations:** 1 Institut de Neuroscience Paris-Saclay (NeuroPSI), UMR 9197 CNRS-Université Paris-Sud, Bât 446, F-91405, Orsay cedex, France; 2 Aix-Marseille University, CRN2M UMR7286 CNRS, 51 Bd Pierre Dramard, 13344, Marseille, France; 3 Dipartimento di Biotecnologie e Scienze della Vita, Università degli Studi dell’Insubria, via J.H. Dunant 3, Varese, Italy; 4 “The Protein Factory”, Centro Interuniversitario di Biotecnologie Proteiche, Politecnico di Milano, ICRM-CNR, Milano, Italy; 5 Università degli Studi dell’Insubria, via Mancinelli 7, Milano, Italy; University of Texas Medical Branch, UNITED STATES

## Abstract

N-methyl-D-aspartate receptors (NMDARs) play a central role in synaptic plasticity. Their activation requires the binding of both glutamate and d-serine or glycine as co-agonist. The prevalence of either co-agonist on NMDA-receptor function differs between brain regions and remains undetermined in the visual cortex (VC) at the critical period of postnatal development. Here, we therefore investigated the regulatory role that d-serine and/or glycine may exert on NMDARs function and on synaptic plasticity in the rat VC layer 5 pyramidal neurons of young rats. Using selective enzymatic depletion of d-serine or glycine, we demonstrate that d-serine and not glycine is the endogenous co-agonist of synaptic NMDARs required for the induction and expression of Long Term Potentiation (LTP) at both excitatory and inhibitory synapses. Glycine on the other hand is not involved in synaptic efficacy *per se* but regulates excitatory and inhibitory neurotransmission by activating strychnine-sensitive glycine receptors, then producing a shunting inhibition that controls neuronal gain and results in a depression of synaptic inputs at the somatic level after dendritic integration. In conclusion, we describe for the first time that in the VC both D-serine and glycine differentially regulate somatic depolarization through the activation of distinct synaptic and extrasynaptic receptors.

## Introduction

N-Methyl-D-aspartate receptors (NMDARs) are central for structural and functional synaptic plasticity as well as cognitive functions [1]. Activation of such receptor requires the binding of both glutamate and a co-agonist [2]. Although glycine was initially identified as the main co-agonist [3,4], subsequent investigations revealed that d-serine, synthesized by serine racemase (SR) [5,6] and present in areas where NMDARs are prevalent [7], would be the preferred co-agonist for synaptic NMDARs [8]. Indeed, enzymatically or genetically induced depletion of d-serine reduces synaptic NMDARs currents and thereby alters synaptic plasticity in the hippocampus [9–12], prefrontal cortex [13], and nucleus accumbens [14]. The role of d-serine at NMDARs is further illustrated by studies showing that synaptic and cognitive impairments during aging is linked to a down-regulation of d-serine synthesis [15]. Detailed analysis of the contribution of the two co-agonists in NMDARs regulation further reveals that in the CA1 area of the mature hippocampus d-serine would preferentially act on synaptic NMDARs whilst glycine would modulate extrasynaptic NMDARs [12] although this segregation has been shown to be developmentally regulated [16]. Alternatively, Li and colleagues (2013) propose that the prevalence of d-serine or glycine at synaptic NMDARs in the lateral nucleus of the amygdala would rather be determined by synaptic activity [17] a scenario also reported for the hippocampus [16]. Despite major progress in the definition of d-serine and glycine functions at excitatory synapses, the nature of the endogenous co-agonist in primary sensory areas like the visual cortex remains to be defined notably during the critical period of enhanced plasticity enabling activity-dependent proper development and maturation of the visual system.

Abundant evidence points to the importance of NMDARs in patterning neuronal networks in the visual cortex [18,19]. Indeed, NMDARs-regulated neurotransmission has been suggested to play an important role in ocular dominance (OD) plasticity in both juvenile and adult rodents [20]. Pharmacological blockade or genetic deletions of NMDARs prevent the OD shift after monocular deprivation (MD) during the critical period or in adult [21,22]. Two recent studies have investigated the functional contribution of d-serine in the plasticity of the visual cortex. Yang and colleagues have shown that d-serine facilitates adult cortical NMDAR-dependent synaptic and OD plasticity in MD mice [23]. Furthermore, d-serine depletion by the enzymatic scavenger d-amino acid oxidase causes NMDAR-dependent phase coupling of otherwise phase-independent gamma generating networks, causing hypersynchrony and a distortion of visual perception [24]. These latter observations suggest that d-serine may serve as an endogenous ligand for VC NMDARs and is necessary for proper visual processing. However, it is still unknown how d-serine and glycine interplay modulates excitatory and inhibitory neuronal networks in the visual cortex.

Here, we use enzymatically-driven depletion of d-serine and glycine levels to ascertain their functions in neurotransmission and synaptic plasticity in acute VC slices of P19-25 old rats. Using whole-cell patch clamp recordings of postsynaptic currents enabling to assess inhibitory and excitatory synaptic conductances at layer 5 pyramidal neurons (L5PyNs), we demonstrate that selective loss of function of d-serine but not glycine reduces synaptic events and prevents induction and expression of NMDAR-dependent inhibitory and excitatory LTP in the VC. Furthermore we show that, in contrast, glycine does not modulate synaptic plasticity *per se* but acts at the dendritic integration level, through the activation of strychnine-sensitive glycine receptors (GlyRs), and thereby controls neuronal gain. The present study therefore shows that, in the VC, control of somatic depolarization depends on synaptic plasticity as such, which requires d-serine, as well as on the modulation of signal integration through the opening of GlyRs.

## Experimental Procedures

### Slices preparation and electrophysiology recordings

Experiments were carried out between September 2010 and December 2014 on male Wistar rats in accordance with the European and Institutional guidelines for the care and use of laboratory animals (Council Directive 86/609/EEC and 2010/63/UE and its application in 2013 by the French National Research Council. Rats were bred under standard conditions in our animal facilitity licensed by the French veterinary service (INSERM U894-CPN; CNPS). Agreement number of the animal house facility is C 91-471-104. Our study includes exclusively in vitro experiments with no in vivo work. The article 3 of the 2010/63/UE directive permits to euthanatize animals by cervical dislocation to excise brain tissues for experiments without any requirement of a specific ethical committee agreement. P21 to P28 rats were killed by cervical dislocation and their brains quickly removed. CNJM, who hold a license (number R-45GRETA-F1-10) delivered by the French veterinary service, sacrificed all animals used in this study.

Wistar male rats aged 21 to 28 days old were subject to the decapitation procedure and after quick removal of the brain, one hemisphere was removed, attached to the stage of a tissue slicer (WPI NVSLM1, U.K) and immersed in ice-cold, oxygenated (i.e., bubbled with 95% O_2_/5% CO_2_) artificial cerebrospinal fluid (ACSF) containing (in mM): 126 NaCl, 26 NaHCO_3_, 10 Glucose, 2 CaCl_2_, 1.5 KCl, 1.5 MgSO_4_ and 1.25 KH_2_PO_4_ (pH 7.5, 310–320 mOsm). Parasagittal slices (250 μm) containing primary visual cortex were obtained and transferred to an holding chamber filled with oxygenated ACSF and maintained at room temperature after an initial 1h incubation at 36°C. For experiments, slices were perfused (2–3 ml/min) at 31°C with oxygenated ACSF. Neurons were patched at 40x magnification using an upright microscope (Zeiss Axioscop FS2+). Patch-clamp recording pipettes (4–5 MΩ) were filled with intracellular solution (in mM): 140 K-gluconate, 10 HEPES, 4 ATP, 2 MgCl2, 0.4 GTP and 0.5 EGTA (adjusted to pH 7.3 with KOH, 280–290 mOsm.kg^-1^). Stable whole-cell voltage-clamp recordings were obtained from layer 5 pyramidal neurons (L5PyNs, identified by the shape of their soma and main apical dendrite and from their firing profile induced by 1s depolarizing steps ranging from 0 to 200 pA) with a Multiclamp 700A amplifier (Axon Instruments, USA). Data were sampled at 2 kHz using a Digidata 1322A acquisition board (Axon Instruments, USA). Voltage data were corrected off-line for a measured liquid junction potential of -10 mV. After capacitance neutralization, bridge balancing was performed on-line under current clamp to make initial estimations of the access resistance (Rs). The latter procedure was repeated before every voltage clamp recording. The membrane input resistance (Rm) and time constant (Ƭ_0_) were determined off line by fitting the mean voltage response to a short hyperpolarizing current pulse applied at rest in current clamp mode. Only cells with a resting potential less than -60 mV and with an access resistance lower than 25 MΩ were considered for analysis. Recordings with more than 25% change in input resistance were also discarded.

### Recording of the NMDA-Excitatory Postsynaptic Currents (EPSCs) in L5PyNs

NMDA-EPSCs were recorded in response to electrical stimulation of layer 2/3 after blockade of GABA_A_ receptors with picrotoxin (100 μM) and AMPA receptors with NBQX (10 μM) at a holding potential of +40 mV using an intracellular solution containing (in mM): Cs-methylsulfonate, 115; HEPES, 10; ATP, 4; CsCl, 20; GTP, 0.4; EGTA, 10 (adjusted to pH 7.4 with CsOH; 281 mOsm.kg^-1^). The origin of the recorded current was confirmed by its complete blockade after application of the NMDA receptor antagonist CPP (1μM) (S1 Fig). At least 5 recordings were stacked and averaged.

### Stimulation protocols

For Excitation–Inhibition (E-I) balance determination, electrical stimulations (10–100 μA, 0.2 ms, 0.05 Hz) were applied in the layer 2/3 of visual cortex using 1 MΩ impedance bipolar tungsten electrodes. Electrodes were positioned in the vicinity of L5PyNs apical dendrite in order to recruit feedforward monosynaptic excitation and inhibition as well as disynaptic inhibition [25]. The stimulation intensity was adjusted in current-clamp [25,26] and set to 2–3 times the amplitude of the stimulation necessary to induce a detectable response in current clamp, which has been shown to generate linear subthreshold postsynaptic responses resulting fom coactivation of excitatory and inhibitory circuits. Under voltage-clamp, 4 to 8 trials were repeated for 4 to 7 holding potentials (depending on the cell resting membrane potential). For LTP induction, theta-burst stimulation (TBS) was applied in layer 2/3 only. TBS consisted of 3 trains of 13 bursts applied at a frequency of 5 Hz, each burst containing four pulses at 100 Hz. Inter-train interval was 10 s. The recording protocol was set as follows: baseline recording of the current responses enabling determination of the different conductances (gT, gE, gI) was performed 10 min after settling of the whole cell patch clamp; then the drug was applied and composite current recordings were resumed pre-TBS as well as 15, 30, 45, and 60 min post-TBS. Pre-TBS recordings were performed while drugs were washing. LTP protocols were applied immediately after the pre-TBS recording.

### Determination of the E-I balance

Data were analysed off-line with Acquis1TM and ElphyTM (Biologic UNIC–CNRS, Gif-sur-Yvette, France). The E-I balance determination is based on the continuous measurement of conductance dynamics during the full-time course of the stimulus-evoked synaptic response. Briefly, we performed post-hoc decomposition of postsynaptic current waveforms in excitatory and inhibitory conductances together with continuous estimation of the apparent reversal potential of the composite responses. This allows a somatic measurement of the E-I balance in L5PyNs after dendritic integration of incoming excitation and inhibition [27].

In order to extract the excitatory and inhibitory conductance changes from the evoked synaptic currents, the neuron is considered as the point-conductance model of a single-compartment cell, described by the following general membrane equation:

$$Cm\frac{dVm(t)}{dt}=gleak(Vm(t)-Eleak)-gE(t)(Vm(t)-Eexc)-gI(t)(Vm(t)-Einh)+Iinj$$

where Cm denotes the membrane capacitance, Iinj the injected current, gleak the leak conductance and Eleak the leak reversal potential. gE(t) and gI(t) are the excitatory and inhibitory conductances, with respective reversal potentials Eexc and Einh.

Evoked synaptic currents were measured and averaged for several (4 to 8) holding potentials. IV curves were then calculated at all time points of the response. In IV curves for every possible delay (t), the value of holding potential (Vh) was corrected (Vhc) from the ohmic drop due to leakage current through the access resistance (Vhc(t) = Vh(t)–I(t) x Rs). An average estimate of the input conductance waveform of the cell was calculated from the best linear fit (mean least square criterion) of the IV curve for each delay (t) following the stimulation onset. Only cells showing a Pearson correlation coefficient for the IV linear regression higher than 0.95 between –90 and –40 mV were considered for calculation of the conductance change in the recorded pyramidal neuron, using the slope of the regression line. The synaptically evoked global conductance term (gT(t)) was then measured by subtracting the resting conductance observed in the absence of stimulation (on a time window of 100 ms before electrical stimulation) from the input total conductance. The synaptic reversal potential of the synaptic conductance (Esyn(t)) was taken as the voltage of the intersection between the IV curve during the synaptic response and the IV curve at rest. Assuming that the evoked somatic conductance change reflects the composite synaptic input reaching the soma, Esyn(t) characterizes the stimulation-locked dynamics of the balance between excitation and inhibition. The global synaptic conductance (gT(t)) was further decomposed into two conductance components (gE(t) and gI(t)) corresponding to the activation of excitatory and inhibitory synapses respectively, each associated with known and fixed reversal potentials. Indeed, we showed [25] that the IV curve in the presence of excitatory transmission blockers (CNQX, D-AP5) is linear between -80 to +10 mV with a reversal potential equal to -80 mV. In the presence of bicuculline, blocking inhibitory inputs on the L5PyNs, the IV curve for excitation is also linear between -80 to +10 mV with a reversal potential equal to 0 mV. Furthermore, D-AP5 had no impact on the evoked current responses recorded at 0.05Hz suggesting that NMDA receptors are not recruited.

Accordingly, the reversal potentials used for the decomposition of the global synaptic conductance were set at 0 mV for excitatory (Eexc) and -80 mV for inhibitory conductance (Einh). In addition, these reversal potentials correspond to values typically found in other studies [28]. Einh corresponds to the reversal potential of GABA_A_ (and not an intermediate value between GABA_A_ and GABA_B_) because in the presence of QX314 in the pipette no variation of the synaptic response was observed [27]. Under our experimental conditions, Esyn(t) took any intermediate values between Eexc (0 mV) and Einh (-80 mV) in such a way that the mathematical conditions of the simplification used to calculate gI(t) and gE(t) were fulfilled. For each component, excitatory and inhibitory, we calculated the conductance change as the mean averaged over a time window of 200 ms. The contribution of each component was expressed by the ratio of its integral value (intgE or intgI) to that of global conductance change (intgT).

Like all somatic recordings, our recordings cannot make rigorous estimates of synaptic events in the distal dendrites, and estimated conductances are ratios of the overall excitatory and inhibitory drive contained in the local network stimulated [29]. However, our measurements are relative changes in conductance magnitude which reflect the cumulative contributions of excitation and inhibition arriving at proximal portions of the neuron. These relative conductance changes at the somatic level define a narrow window over which input integration and spike output can occur [30].

### Determination of the time constants and electrotonic length

Membrane time constants (Ƭ_0_) were determined by analyzing the time course of the membrane voltage deflection according to the method described by Rall [31]. This method consists in “peeling exponentials” by plotting the natural log of the response expressed as a percentage of the peak negative potential. In our recordings, the late portion of the charging phase (for time points between 5 and 15 ms) always fitted a linear regression of which the slope was equal to -1/Ƭ_0_. For each cell and hyperpolarizing intensity, the membrane time constant was thus calculated using this equation. Rall’s method also allows determination of the equalizing time constant (Ƭ_1_), which represents the time required for the current to spread to the proximal dendrites. Ƭ_1_ is given by the second order exponential observed for time points earlier than 5ms. This value was obtained by plotting the difference between the membrane time constant regression line from the points lying above this line, normalizing the y-intercept of this new line to 100% and reading Ƭ_1_ as the negative inverse of the slope. From two time constants, the somatodendritic electrotonic length (L) was estimated using the relation: L = π (Ƭ_0_/ Ƭ _1_)^-1/2^.

### Statistical analysis

Data reported are mean ± standard error of the mean (SEM) of n cells. Each individual cell was recorded in a single acute slice (3–4 slices/ animal per day) in order to avoid repeated exposure to the TBS protocol and/or drug application. When a LTP protocol was applied (TBS), post-TBS conductance integral values were normalized to pre-TBS conductance integrals (100%) and expressed as percentage of baseline. LTP was defined as a change >130% of baseline 1 h post-TBS. Statistics were performed using the InVivoStat software (Mockett Media). Paired samples for IntgT, IntgE, IntgI, between the control condition (before TBS) and given times after TBS (15, 30, 45 or 60 min) were analyzed using the paired student *t*-test.

### Chemicals and enzymes

All chemicals used for electrophysiology experiments were obtained from Sigma-Aldrich except NBQX which were from Ascent Scientific (Bristol UK). Appropriate stock solutions were made, stored at −20°C and diluted to the final concentration in aCSF.

The selective depletion of D-serine and glycine was obtained by using recombinant *Rg*DAAO (EC 1.4.3.3) and recombinant *Bs*GO (EC 1.4.3.19) which were overexpressed in *Escherichia coli* cells and purified as previously reported [32,33]. The final enzyme preparations displayed the following activity: *Rg*DAAO = 100 ± 15 U/mg protein on d-serine as substrate; *Bs*GO = 0.9 ± 0.2 U/mg protein on glycine as substrate. These flavoenzymes specifically degrade d-serine (*Rg*DAAO) and glycine (*Bs*GO), as demonstrated by the corresponding apparent kinetic efficiency (*k*_cat_/*K*_m_ ratio) values: *Rg*DAAO *k*_cat_/*K*_m_ ratios were 3.0 and 0.058 mM^−1^ s^−1^ [34], while those determined for *Bs*GO were 0.00025 and 0.867 mM^−1^ s^−1^ on d-serine and glycine, respectively [33]. Enzymatic treatments were performed by incubating for at least 45 min and then continuously perfusing the slices with ACSF containing *Rg*DAAO (0.2 U/ml) or *Bs*GO (0.1 U/ml). An inactive variant of *Rg*DAAO (Δ*Rg*DAAO) that does not dehydrogenate d-amino acids due to the substitution of the pivotal active site residue Arg285 with Alanine [35] was used as control. Enzymes activity is not altered by the presence of picrotoxin (100 μM) and NBQX (10 μM) during EPSCs recordings [12,16].

### Immunohistochemistry

Rats were deeply anesthetized with pentobarbital (6%) and perfused transcardially with phosphate buffer (PB) 0.1 M (pH 7.3) and paraformaldehyde (4%) supplemented with 0.25% glutaraldehyde. The brain was post-fixed overnight in the same solution and finally cryoprotected with 30% sucrose in 0.1M PB. Brains were sliced (30 μm) using a vibratome and immunostainings were performed as previously described [13]. Immunostained sections were observed with a ZEISS LSM 710 confocal microscope (ZEN software) with 405 diode, 488 and 633 laser lines. All image acquisitions were achieved using 40X and 60X magnification and variable numerical zooms. Channels were acquired with sequential scanning with a pinhole aperture of 1 airy unit each. Images were then analyzed using ImageJ software. Staining specificity was verified with negative controls in which primary or secondary antibodies were omitted. Affinity-purified primary antibodies were: mouse monoclonal anti serine racemase (Transduction Laboratories: 1/1000), rabbit polyclonal anti-d-serine (GemacBio: 1/1000), mouse polyclonal anti-GFAP (Sigma: 1/2000, clone G-A-5), mouse monoclonal anti-glyR (mAb4a, Synaptic Systems 1/100). Secondary antibodies were obtained from Invitrogen: Alexa 488-Goat anti-mouse (1/1000), Alexa 633-Goat anti-rabbit (1/1000) and from Life Technologies: FITC conjugated mouse antibody (1/400).

## Results

### d-Serine is the endogenous agonist of synaptic NMDARs in the VC

**Fig 1:**
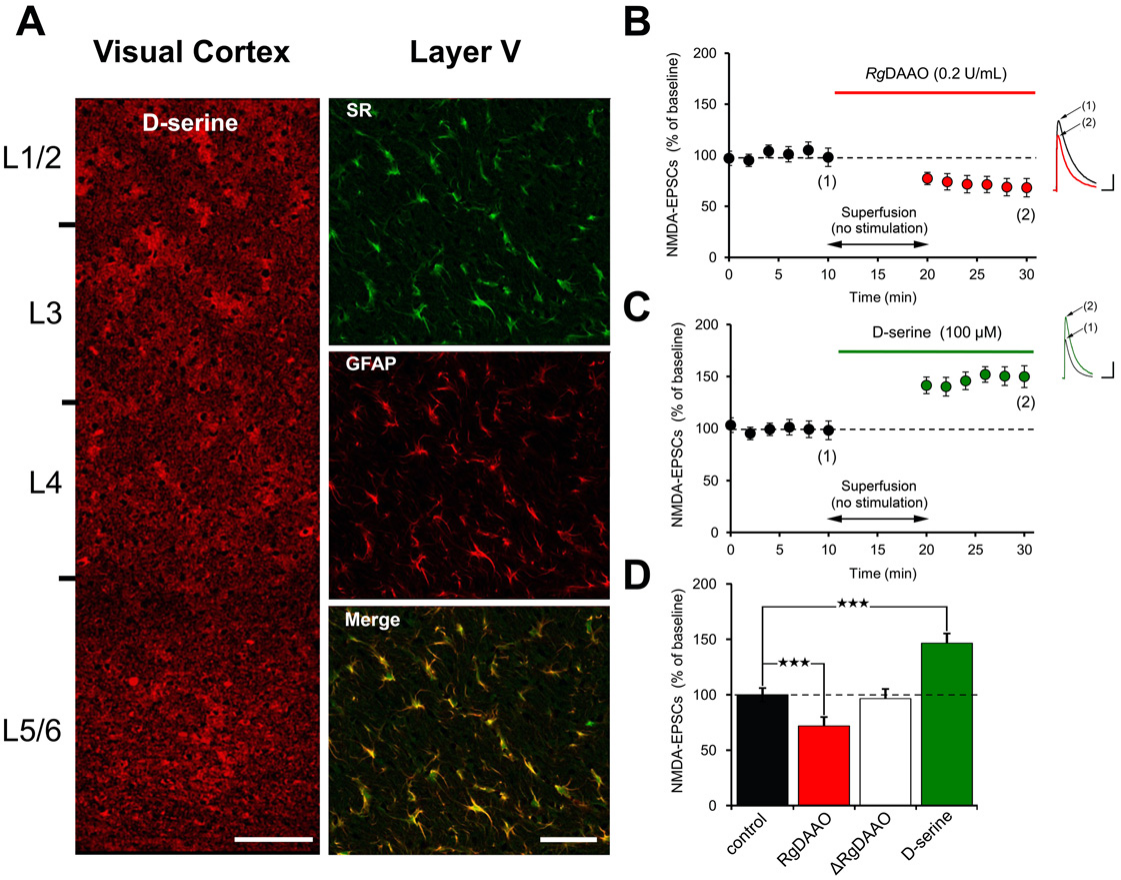
D-serine modulates synaptic NMDARs current at VC L5PyNs **A:** Immunofluorescence for D-serine, and GFAP revealed that D-serine is expressed across all layers of P22-25 (5 slices, 3 animals) VC. The D-serine producing enzyme serine racemase (SR) was also found to be expressed and co-localized with the astroglial marker GFAP. Images are Z-stack of 10 serial confocal images with a thickness of 1μm. Scale bars: left, 200 μm; right, 50μm **B-D:** Applications of the D-serine degrading enzyme *Rg*DAAO (0.2 U/ml) reduces synaptically evoked NMDA-EPSCs (n = 5 cells, 5 slices, 2 animals) (B) while its inactive form Δ*Rg*DAAO has no effect (n = 5 cells, 5 slices, 2 animals) (D). Scale bars: 100pA, 500ms. Conversely D-serine (100 μM) significantly potentiates NMDA-EPSCs (n = 5 cells, 5 slices, 2 animals) (C) Scale bars: 200pA, 500ms. ***p<0.001.

We first assessed the putative contribution of d-serine in the VC by immunostainings for d-serine. We found D-serine and the D-serine producing enzyme serine racemase to be present in all layers including layer 5 of the VC (Fig 1A), at P22-P25 suggesting that d-serine may already contribute to synapse and neuronal networks functions during the first weeks of postnatal development. We then assessed the functions of d-serine in driving synaptic activity by recording postsynaptic NMDA currents (NMDA-EPSCs) in L5PyNs. Bath application of the selective d-serine scavenger d-amino acid oxidase *(Rg*DAAO, 0.2 U/ml) [13,16] decreased NMDA-EPSCs by 29.3 ± 5.1% (Fig 1B and 1D; n = 5 cells, 5 slices, 2 animals P<0.001) to a similar order of magnitude as the co-agonist site blocker 7-Cl-KYN (S1 Fig), while the inactive variant of *Rg*DAAO (Δ*Rg*DAAO) had no effect (Fig 1D). Conversely, bath application of d-serine (100 μM) increased NMDA-EPSCs by 46.3 ± 7.5% (Fig 1C & 1D; n = 5 cells, 5 slices, 2 animals, P<0.001) thus showing that the co-agonist site of NMDARs is not saturated in the VC, at least during the time of critical period for plasticity.

**Fig 2:**
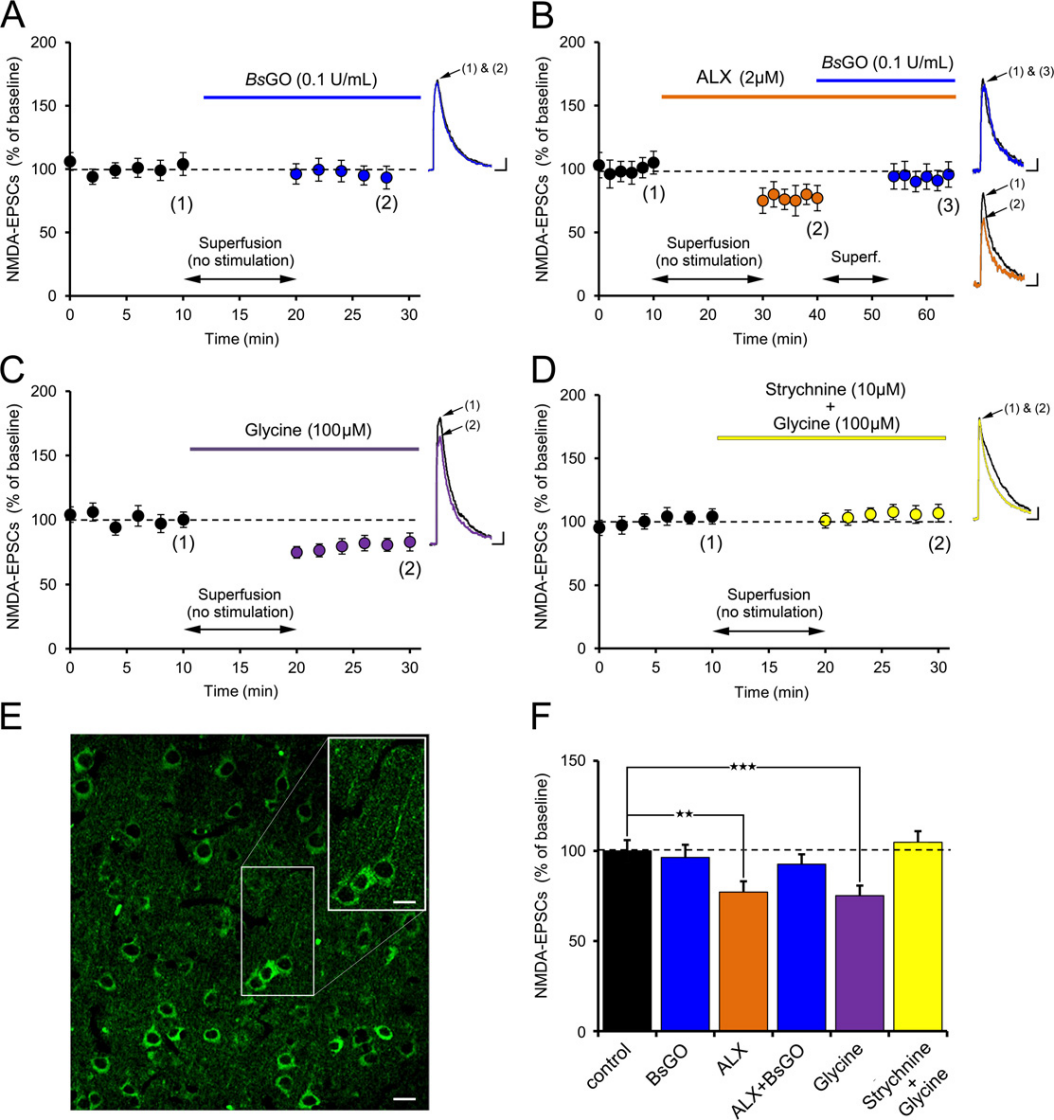
Glycine is not the endogenous co-agonist of L5PyNs VC NMDARs **A:** Application of the glycine degrading enzyme *Bs*GO (0.1 U/ml) has no effect on NMDA-EPSCs (n = 5 cells, 5 slices, 2 animals), indicating that glycine is not the endogenous co-agonist of synaptic L5PyrNs VC NMDARs. Scale bars: 100pA, 500ms. **B:** Further, enhancing endogenous glycine levels with the glycine transporter blocker ALX5407 (2 μM) decreased the NMDARs response, an effect blocked by *Bs*GO (n = 4 cells, 4 slices, 2 animals). Scale bars: 25pA, 250ms. **C:** A similar result is obtained by exogenous application of glycine (100μM) (n = 5 cells, 5 slices, 2 animals). Scale bars: 50pA, 500ms. **D:** Such downregulation of NMDA-EPSCs by glycine is remarkably blocked by the glycinergic receptors (GyRs) antagonist strychnine (10μM) (n = 5 cells, 5 slices, 2 animals). Scale bars: 50pA, 500ms. **E:** Immunofluorescence for GlyRs revealed that, in the VC, they are mainly expressed in L5PyRNs notably at the somatic and dendritic level. Scale bar: 50μm, inset: 30μm. **F:** Altogether, these results indicate that glycine downregulates NMDA-EPSCs through activation of GlyRs ** p<0.01, *** p<0.001.

Because *Rg*DAAO only partially reduced the amplitude of NMDA-EPSCs (Fig 1B & 1D), we then addressed whether glycine in addition to d-serine could also be a co-agonist for synaptic NMDARs in VC of young rats. Depletion of endogenous glycine level using the recombinant *Bacillus subtilis* glycine oxidase (*Bs*GO, 0.1U/ml) did not affect NMDA-EPSCs amplitude (Fig 2A & 2F; n = 5 cells, 5 slices, 2 animals, P>0.05). Because such lack of effect could be due to failure of *Bs*GO to reduce glycine levels [13,16,36] we performed control experiments with ALX5407 (2 μM) to increase endogenous glycine levels by inhibiting glycine transporters type 1 (GlyT1) and then applied *Bs*GO (Fig 2B). ALX5407 decreased rather than increased NMDA-EPSCs amplitude by 20.3 ± 3.1% (Fig 2B & 2F; n = 4 cells, 4 slices, 2 animals, P<0.01), an effect indeed reversed by *Bs*GO (Fig 2B & 2F) then showing that the latter was effective in depleting endogenous glycine. Strikingly, bath application of glycine (100μM) significantly decreased NMDA-EPSCs amplitude by 21.9 ± 4.2% (Fig 2C & 2F; n = 5 cells, 5 slices, 2 animals, P<0.001). These observations indicate that d-serine rather than glycine is the co-agonist of synaptic NMDARs in the VC of young rats under normal conditions.

Insofar as *Bs*GO did not alter NMDA-EPSCs, we hypothesized that the down neuromodulation exerted by glycine on excitatory neurotransmission may occur through activation of strychnine-sensitive glycine receptors (GlyRs), as seen in the hippocampus [37]. To test this hypothesis, we blocked GlyRs with bath application of 10 μM strychnine and showed that such treatment was sufficient to prevent downregulation of NMDA-EPSCs by 100 μM glycine (Fig 2D & 2F; n = 5 cells, 5 slices, 2 animals). Besides, we verified the presence of these receptors by performing immunostainings for GlyRs [38]. Fig 2E shows that GlyRs are indeed abundant in apical dendrites and soma of pyramidal neurons indicating their possible role in integrating information at L5PyNs of VC.

Altogether, these data support that in L5PyNs of VC, d-serine regulates synaptic NMDARs efficacy by acting as the co-agonist of NMDARs, while glycine acts downstream through activation of dendritic and somatic GlyRs.

### d-Serine enables visual cortex LTP

**Fig 3:**
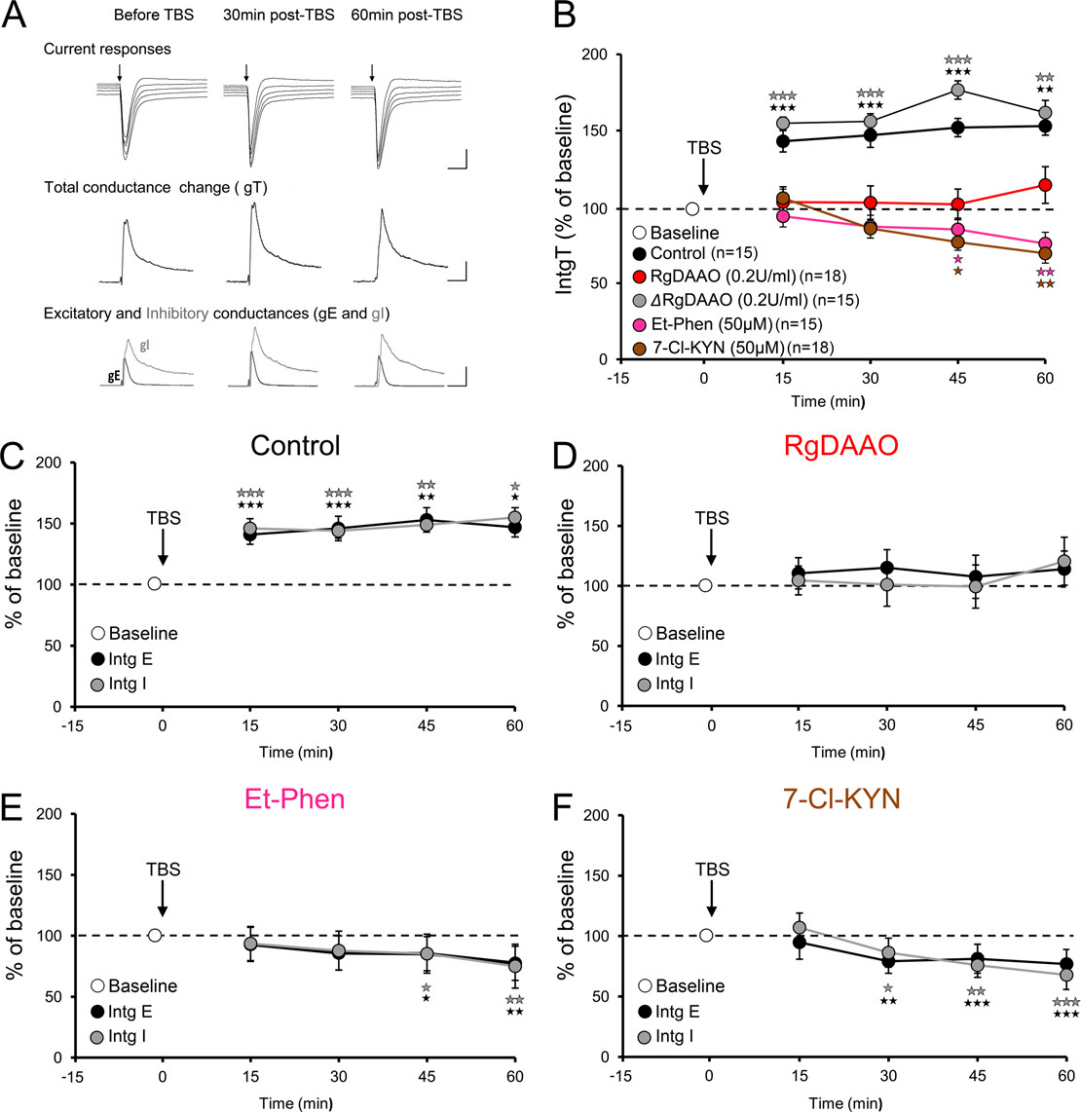
D-serine is required for VC long-term potentiation **A:** Upper panel shows representative composite current responses of L5PyN for the range of imposed potentials before and during LTP. Scale bars: 300pA, 50ms. Medium panels displays the corresponding total conductance change gT before and during LTP. Lower panels show decomposition of gT into excitatory (gE, black) and inhibitory (gI, grey) conductances (n = 15 cells, 15 slices, 8 animals). Scale bars: 4nS, 50ms. **B:** Changes in the gT integral (IntgT) calculated every 15 min, up to 1 h post-TBS, show that LTP is abolished by blocking the co-agonist binding site of NMDARs with 7-Cl-KYN, removing D-serine through the D-serine degrading enzyme *Rg*DAAO (n = 18 cells, 18 slices, 9 animals) or preventing D-serine production via blockade of the D-serine producing enzyme serine racemase with Et-Phen (n = 17 cells, 17 slices, 8 animals). This indicates that D-serine is required for VC L5PyNs LTP. **C-F:** Excitatory (black) and inhibitory (grey) conductances were found to be equally affected by TBS application, regardless of the treatment, indicating that the E-I balance is unaltered by D-serine and LTP. *p<0.05, **p<0.01, ***p<0.001 compared to pre-TBS.

Having established that d-serine is the endogenous ligand of NMDARs in the young visual cortex, we next examined its contribution to long-term potentiation (LTP) by determining the total synaptic conductance change (referred as IntgT) and by analyzing both excitatory (eLTP) and inhibitory (iLTP) components of the response. We have previously shown that Theta Burst Stimulation (TBS) of layer 2–3 of the VC resulted in similar levels of eLTP and iLTP recorded at the soma of L5PyNs and that this form of LTP depends on NMDARs functions [25]. Accordingly, in the present study, TBS protocol induced comparable LTP (Fig 3A & 3B; IntgT: 148.7 ± 3.0% of baseline, Ps<0.001) and resulted in similar levels of eLTP and iLTP (Fig 3B & 3C; IntgE: 146.7 ± 4.0% of baseline, IntgI: 150.1 ± 3.0% of baseline, Ps<0.001, n = 15 cells, 15 slices, 8 animals), hence not modifying the E-I balance. Most interestingly, bath application of the NMDARs co-agonist site blocker 7Cl-KYN (50 μM) [39] during and after TBS protocol not only prevented LTP but depressed synaptic responses after TBS administration (IntgT: 68.5 ± 6.3% of baseline, P<0.001; n = 18 cells, 18 slices, 9 animals) at both excitatory and inhibitory level (Fig 3B & 3F; IntgE: 76.8 ± 6.2% of baseline, P<0.01; IntgI: 67.7 ± 6.8% of baseline, P<0.001). TBS induced LTP is therefore, in the VC, NMDA-receptor dependent, as expected from our previous work [40].

Accordingly, depletion of endogenous D-serine with *Rg*DAAO (0.2 U/ml) prevented induction of LTP (Fig 3B & 3D, IntgT:105.4 ± 8.3% of baseline; IntgE:111.8 ± 10.8% of baseline; IntgI: 106.4 ± 10.4% of baseline, n = 8 cells, 8 slices, 4 animals, Ps>0.05) while application of its inactive form Δ*Rg*DAAO throughout the experiment showed no effect (Fig 3B; IntgT: 154.8 ± 12.9% of baseline; IntgE: 144.3 ± 10.5% of baseline; IntgI: 162.1 ± 16.2% of baseline; n = 15 cells, 15 slices, 8 animals, Ps<0.001), thus confirming the specificity of *Rg*DAAO blockade.

Interestingly, in contrast with 7Cl-KYN, *Rg*DAAO failed to engender TBS-induced synaptic depression. Since this apparent discrepancy could reflect incomplete depletion of endogenous d-serine by *Rg*DAAO we further tested the contribution of d-serine in LTP using phenazine Ethosulfate (Et-Phen), an inhibitor of serine racemase, throughout the experiment [41–43]. Pharmacological inhibition of SR not only prevented the TBS-induced eLTP and iLTP as observed with *Rg*DAAO (Fig 3B & 3E) but also induced depression of the synaptic responses, as observed with 7Cl-KYN (Fig 3B & 3E; IntgT: 75.9 ± 7.4% of baseline; IntgE: 77.3 ± 7.6% of baseline; IntgI: 75.3 ± 8.6% of baseline; n = 17 cells, 17 slices, 8 animals, Ps<0.01). Altogether, the dataset therefore indicates that d-serine is required for both eLTP and iLTP and that its absence unmasks a depression of evoked synaptic responses recorded at the soma of L5PyNs.

### Glycine downregulates current spread in VC L5PyNs

**Fig 4:**
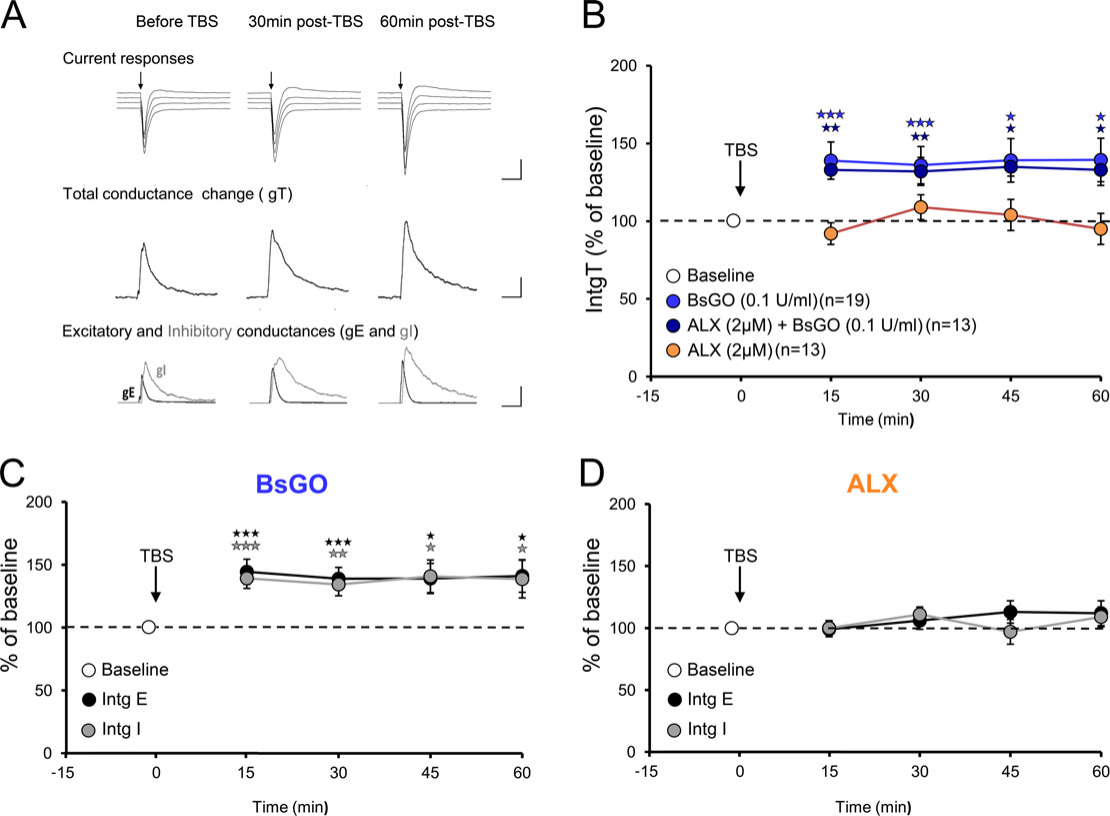
Increasing endogenous glycine level prevents the induction of LTP. **A:** Upper panel shows representative composite current responses of L5PyN for the range of imposed potentials before and during LTP. Scale bars: 150pA, 50ms. Medium panels displays the corresponding total conductance change gT before and during LTP. Lower panels show decomposition of gT into excitatory (gE, black) and inhibitory (gI, grey) conductances. Scale bars: 4nS, 50ms. **B:** Changes in IntgT up to 1 h post-TBS show that LTP is abolished when endogenous glycine levels are increased by blocking the glycine transporter with ALX (2μM) (n = 13 cells, 13 slices, 6 animals). The glycine degrading enzyme *Bs*GO has no effect on LTP (n = 19 cells, 19 slices, 9 animals), and prevents the effect of ALX (n = 13 cells, 13 slices, 6 animals), thus confirming that the latter is attributable to endogenous glycine rise. **C-D:** Excitatory (black) and inhibitory (grey) conductances were found to be equally affected by TBS application, regardless of the treatment, indicating that the E-I balance is unaltered by glycine and LTP. *p<0.05, **p<0.01, ***p<0.001 compared to pre-TBS.

Given the lack of effect of *Bs*GO on NMDA-EPSCs and their downregulation in the presence of glycine, we then sought to ascertain to which extent endogenous glycine may or not contribute to the modulation of L5PyNs synaptic current during higher regime of activity. To this end, glycine depletion was achieved again by application of *Bs*GO (0.1 U/ml) throughout the experiment. Under these conditions, LTP was found to be unchanged (Fig 4B & 4C; IntgT: 136.4 ± 6.4% of baseline; IntgE: 139.8 ± 9.7% of baseline; IntgI: 134.2% ± 9.0% of baseline; n = 19 cells, 19 slices, 9 animals, Ps<0.01) indicating that unlike d-serine, reduced level of glycine does not limit LTP induction and expression in the rat VC. We also tested whether, conversely, increase in endogenous glycine could affect LTP. To this end we applied the selective blocker of GlyT1 ALX5407 (2μM) half an hour before TBS and throughout the experiment [36] in order to build up glycine concentration in the cortical slice. Under ALX5407, TBS failed to induce LTP (Fig 4B & 4D; IntgT: 101.2 ± 8.0% of baseline; IntgE: 110.7 ± 9.3% of baseline; IntgI: 104.2% ± 7.4% of baseline, Ps>0.05 n = 13 cells, 13 slices, 6 animals). We further confirmed that this effect was attributable to the action of glycine as adding *Bs*GO (0.1 U/ml) in the presence of ALX5407 (2μM) throughout the experiment showed no effect on VC LTP (Fig 4B; IntgT: 133.2 ± 4.8% of baseline, IntgE: 135.4 ± 6.2% of baseline and IntgI: 131.1 ± 4.0% of baseline respectively; Ps> 0.01, n = 13 cells, 13 slices, 6 animals). This result was identical to the result obtained in the presence of *Bs*GO alone. Since *Bs*GO was effective in depleting glycine, we conclude that raising endogenous glycine concentration prevents somatic recording of LTP in L5PyNs.

**Fig 5:**
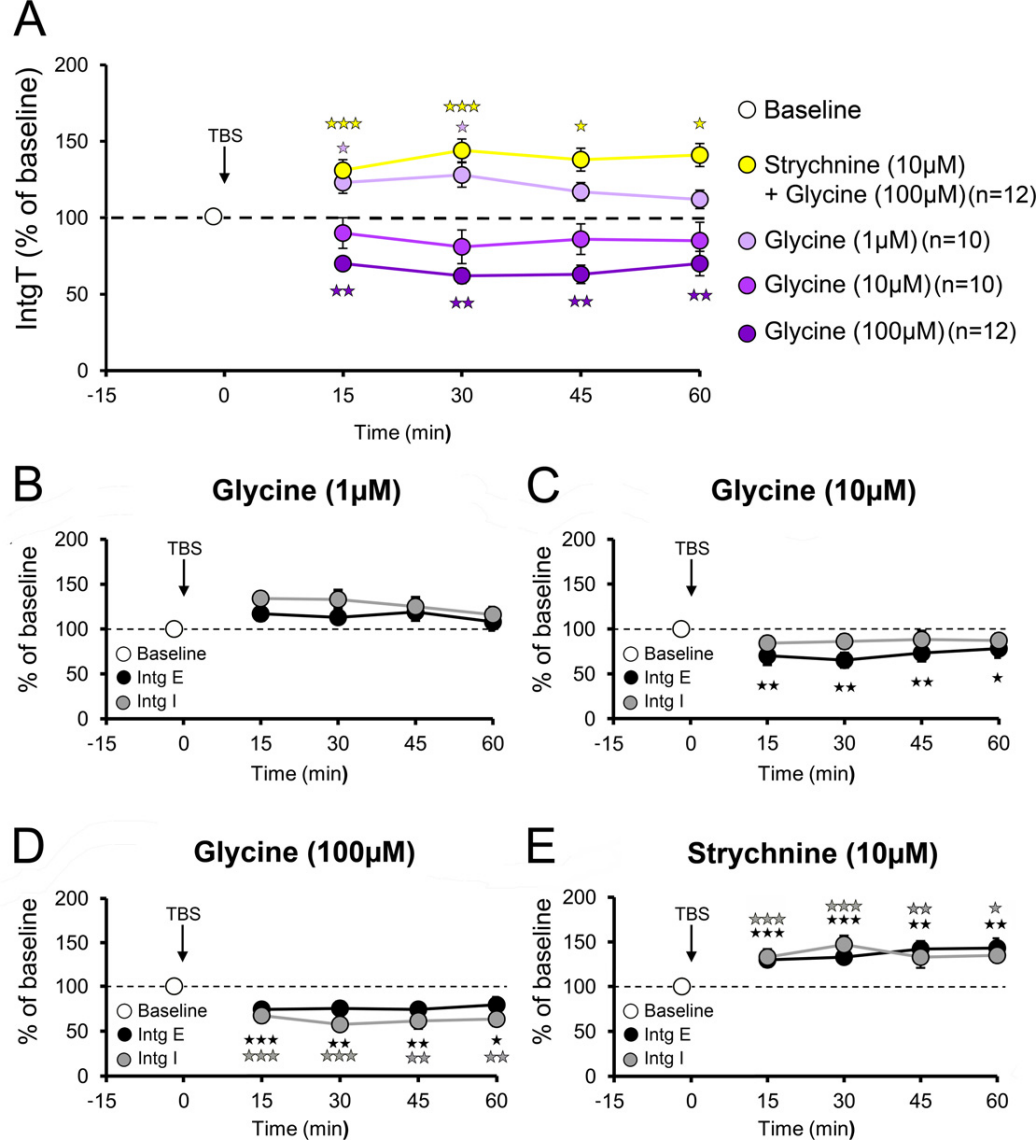
Dose effects of various concentrations of glycine on LTP **A:** At the concentration of 1μM glycine the initial potentiation induced by TBS does not last 1 h, thus indicating that at such low concentration is enough to prevent LTP (n = 10 cells, 10 slices, 5 animals). At 10μM glycine not only prevents all potentiation but also slightly decreases conductances recorded at the soma (n = 10 cells, 10 slices, 5 animals), although this reduction is not significant. At 100μM glycine blocks LTP and induces a significant depression up to 1 h post-TBS (n = 12 cells, 12 slices, 6 animals). **B-E:** Excitatory (black) and inhibitory (grey) conductances were found to be equally affected by TBS application, regardless of the treatment, confirming that the E-I balance is unaltered by glycine and LTP. *p<0.05, **p<0.01, ***p<0.001 compared to pre-TBS.

To further characterize the dose-dependent effect of glycine on TBS induced plasticity, we measured the effect of three concentrations of glycine on excitatory and inhibitory plasticity. Application of 1 μM glycine was sufficient to annihilate LTP (Fig 5A & 5B; IntgT: 120.2 ± 8.1% of baseline; IntgE: 114.4 ± 8.8% of baseline; IntgI: 127.3 ± 7.5% of baseline, n = 10 cells, 10 slices, 5 animals, P>0.05). At higher concentrations, exogenous glycine (10 μM) resulted in a weak depression following TBS (Fig 5A & 5C; IntgT: 84.5 ± 6.2% of baseline; IntgE: 81.6 ± 7.6% of baseline; IntgI: 85.5 ± 7.9% of baseline, n = 10 cells, 10 slices, 5 animals, Ps> 0.05), an effect significantly amplified at 100 μM (Fig 5A & 5D; IntgT: 66.8 ± 5.7%; IntgE: 74.4 ± 7.2% of baseline; IntgI: 62.2 ± 6 .4% of baseline; n = 12 cells, 12 slices, 6 animals, Ps<0.001). Insofar as we found that glycine is able to downregulate NMDARs currents through activation of GlyRs, we analyzed contribution of the latter by applying TBS in the presence of glycine (100 μM) and the GlyR blocker strychnine (10 μM). This resulted in an LTP comparable to that obtained in control conditions (Fig 5A & 5E; IntgT: 138.9 ± 6.7% of baseline; IntgE: 137.3 ± 7.9% of baseline; IntgI: 140.7% ± 6.7% of baseline; n = 12 cells, 12 slices, 6 animals, Ps> 0.01). Furthermore, applying TBS whilst blocking the NMDARs co-agonist site with 7-Cl-KYN in the presence of glycine (100μM) still depressed synaptic responses (S2 Fig; IntgT: 73.9 ± 11.4% of baseline; IntgE: 77.1 ± 10.3% of baseline; IntgI: 62.5 ± 12.3% of baseline; n = 4, Ps<0.05) indicating that NMDARs are not involved in these downregulations. Finally, in the presence of 7-Cl-KYN and strychnine TBS induced no change (S2 Fig; IntgT: 101.2 ± 10.9% of baseline; IntgE: 103.5 ± 12.7% of baseline; IntgI: 91.5 ± 10.2% of baseline; n = 4; Ps>0.05) in current magnitude, thus implying that GlyRs activation underlies the reduction of recorded currents. We therefore conclude that increased levels of glycine during high regime of activity such as TBS activate GlyRs and eventually leads to a depression of the conductances recorded after TBS at the somatic level. Given the extrasynaptic dendritic and somatic localisation of GlyRs, we hypothesized that their activation results in a shunt of synaptic inputs.

**Fig 6:**
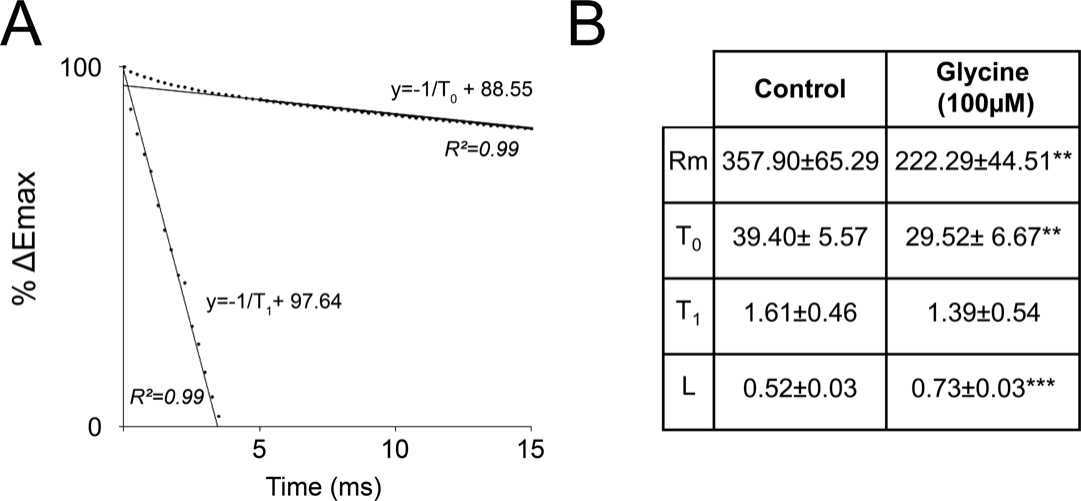
Glycine reduces VC L5PyNs dendritic current spread **A:** Membrane time constants Ƭ_0_ and Ƭ_1._ were calculated by plotting the natural log of the response expressed as percentage of the peak negative potential (% ΔEmax) to “peel” the first order exponential for time points lying between 5 and 15ms. Ƭ_0_ could then be read as the slope negative inverse of the regression line. The second order exponential for time points earlier than 5ms was peeled by plotting the difference between the Ƭ_0_ regression line from the points lying above this line and normalizing the y-intercept of the Ƭ_1_ regression line to 100%. Ƭ_1_ could then be read as the slope negative inverse of the normalized Ƭ_1_ regression line. **B:** Calculation of the electrotonic length (L) using the equation L = π (Ƭ_0_/ Ƭ _1_)^-1/2^ showed that L is significantly increased in the presence of 100μM glycine, indicating that the dentritic current spread attenuation is higher in the latter case. **p<0.01,***p<0.001.

To assess such possibility, we calculated the electrotonic length of L5PyN and used it as an index of current spread efficacy according to the method described by Rall [31]. Remarkably, the electrotonic length was found to be significantly higher in the presence of 100 μM glycine (Fig 6B; Control: 0.52 ± 0.03, n = 15; Glycine: 0.73 ± 0.03, n = 16 cells, 16 slices, 8 animals; P<0.01), an effect that corresponds to an increased attenuation of the distal currents reading at the somatic level [44,45]. We conclude that activation of dendritic GlyRs results in a filtering effect. In all, these data indicate that, in the VC, activation of GlyRs by elevated endogenous glycine levels during high regime of activity filters synaptic inputs conveyed through the dendritic tree, thereby masking LTP induced at distal synapses and resulting in LTD-like changes in current readings at the somatic level.

## Discussion

The present study provides evidence that d-serine controls NMDAR-dependent LTP in VC L5PyNs whilst glycine influence neurotransmission at a different level, by activating extrasynaptic GlyRs distributed along the apical dendrite. Activation of the GlyRs when the concentration of glycine increases indeed results in a shunting inhibition of afferent inputs which thus display a depression (a LTD-like effect) instead of an LTP at the soma after dendritic integration.

Whilst former investigations have supported that glycine is the co-agonist of NMDARs [3,4,46,47], recent work have shown that reducing d-serine levels impairs NMDAR-mediated processes in several structures, including the hippocampus, prefrontal cortex, nucleus accumbens or amygdala [9,10,12–14,17,48], suggesting that d-serine is likely to be the co-agonist for synaptic NMDAR prevailing in many brain areas, even though glycine remains engaged in the modulation these receptors [11, 16, 17]. Using D-serine and/or glycine, several reports indicate that the co-agonist site of synaptic NMDARs is not saturated in the VC of cats and rats, during and after the critical period of plasticity [49–52]. However, thus far, identity of the prevalent co-agonist remained undetermined in this structure. Our data show that, at VC L5PyNs, reducing d-serine function using a variety of pharmacological treatments prevents the induction of LTP whilst depleting glycine has no effect, thus demonstrating in the VC that d-serine and not glycine is the dominant endogenous ligand of synaptic NMDARs, as seen in other brain areas [9,12,13,16,53]. It however remains possible that modulation of extrasynaptic NMDARs is different and involves glycine.

Both d-serine and glycine have been reported to be present in the micromolar range in various cortical areas [7,54], although their concentrations may differ between the *in vivo vs ex vivo* situation. Insofar as our results indicate that glycine does not undertake the main role of NMDA receptor co-agonist, we further investigated its role in this brain region. The presence of GlyRs along the dendritic tree of L5PyNs suggests a functional role at this level. Interestingly however, removing glycine from the preparation using *Bs*GO throughout the experiment showed no effect on NMDA receptor function. Thus, considering the inhibitory effect we observed on NMDA currents and the increase in electrotonic length engendered by glycine application, we propose that by opening GlyRs, rise of glycine that occurs primarily during high regime of activity would allow for a potent filtering of synaptic inputs conveyed through the neuron. Although membrane depolarizations may be large and sharp locally at the synapse, the resulting changes in somatic potential are much slower and smaller after dendritic propagation [55]. Insofar as opening of chloride permeability at resting potential mainly acts as a shunt since the equilibrium potential for Cl^-^ is -80mV [25], activation of non-synaptic dendritic GlyRs should indeed accentuate current attenuation upon propagation. Such interpretation is supported by our observation that downregulation of NMDA currents and LTP by glycine is fully prevented by the GlyRs blocker strychnine. A similar effect has also been observed in the hippocampus where GlyRs act as a tonic shunt, thereby depressing excitatory neurotransmission and inducing LTD [37,56,57]. The present study thus shows that the situation is most likely similar in the VC, where EPSCs arising at the synaptic level are filtered and depressed along the dendritic tree of L5PyNs upon opening of GlyRs, this results in a LTD-like plasticity at the soma. Such selective activation of GlyRs and not NMDARs in the VC is most likely attributable to the differential sites of action of these receptors. It is indeed readily conceivable that glycine remains confined to extrasynaptic sites, where GlyRs are expressed, as glycine transporters can prevent its access to synaptic confinements, as found in the hippocampus [12]. In the cortex and hippocampus, GlyRs have been shown to be primarily expressed at somatic and dendritic extrasynaptic sites [58,59]. These anatomical observations are also corroborated by a large body of physiological investigations reporting that synaptic currents are abolished by antagonists of glutamate and GABA receptors in the postnatal cortex, including our work in the VC [60,61], and thereby suggesting that cortical GlyRs are not synaptically activated. Such data support the early demonstration by Flint and colleagues that GlyRs are activated non-synaptically [62]; consistent with this finding, a recent report found that application of GlyR antagonist strychnine dampens membrane currents, without affecting spontaneous synaptic events [63]. In all, our findings thus represent a new framework integrating the complex functions of d-serine and glycine in the modulation of synapse activity and the dynamics of VC neuronal networks.

The brain uses a variety of strategies to process and extract information upon the large range of sensory inputs it receives. Several investigations have sought the optimal strategies the brain may employ to tune the best signal-to-noise ratio obtainable, such as, for example, gain control [64]. Yet, in primary sensory cortices, the strategies to scale integration of the input strength and extract relevant information from noise remain poorly understood [65,66]. One interesting model postulates that the VC would select pertinent information by a noise-filtering action, dampening responses to irrelevant visual noise [67]. Remarkably, a recent study provides fMRI data supporting the latter hypothesis [68]. By demonstrating that activation of GlyRs along the L5PyNs dendrite enables efficient shunt of synaptic inputs while maintaining the E-I balance intact, the present study shed light on a mechanism that likely participates in the selective wave attenuation of sensory inputs, known to be necessary for the adaptation of inputs intensity to visual processing [69].

## Supporting Information

S1 Fig NMDAR currents are antagonized by CPP and 7-Cl-KYN. A: Administration of the selective NMDARs antagonist CPP (1μM) expectedly abolished the recorded current, thus confirming their nature (n = 4). **B:** Bath application of the co-agonist site blocker 7-Cl-KYN decreased NMDA-EPSCs to the same extent as the selective D-serine scavenger D-amino acid oxidase thus suggesting that D-serine is the endogenous co-agonist of NMDARs in the visual cortex (n = 3).
(TIF)

S2 Fig TBS induced depression depends on GlyRs and not NMDARs. **A:** The depression observed after TBS administration in the presence of glycine 100μM is not affected by the NMDAR co-agonist binding site blocker 7-Cl-KYN (50μM) indicating that putative regulation of NMDAR by glycine does not play a role in this process (n = 4). **B:** Instead, GlyRs underlie such downregulation as the GlyRs blocker strychnine (10μM) abolishes the TBS induced depression (n = 4). *p<0.05, **p<0.01, ***p<0.001.
(TIF)
