# Supplementary figures and images for: D-Serine and Glycine Differentially Control Neurotransmission during Visual Cortex Critical Period
Source: PLoS One. 2016 Mar 22;11(3):e0151233. doi: 10.1371/journal.pone.0151233 (PMC4803205; doi:10.1371/journal.pone.0151233)

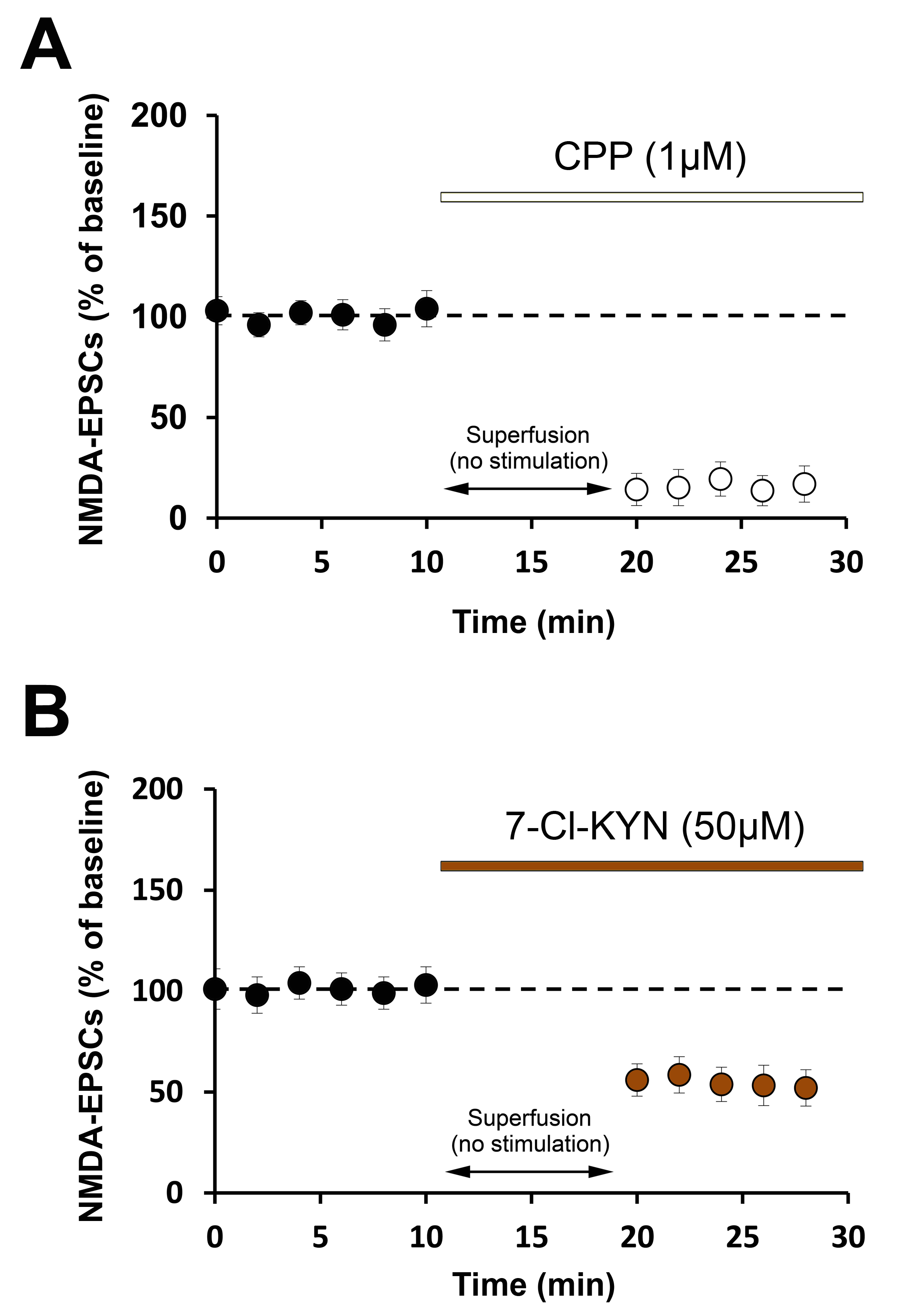

Supplement: S1 Fig — A: Administration of the selective NMDARs antagonist CPP (1μM) expectedly abolished the recorded current, thus confirming their nature (n = 4). B: Bath application of the co-agonist site blocker 7-Cl-KYN decreased NMDA-EPSCs to the same extent as the selective D-serine scavenger D-amino acid oxidase thus suggesting that D-serine is the endogenous co-agonist of NMDARs in the visual cortex (n = 3). (TIF) [file pone.0151233.s001.tif]

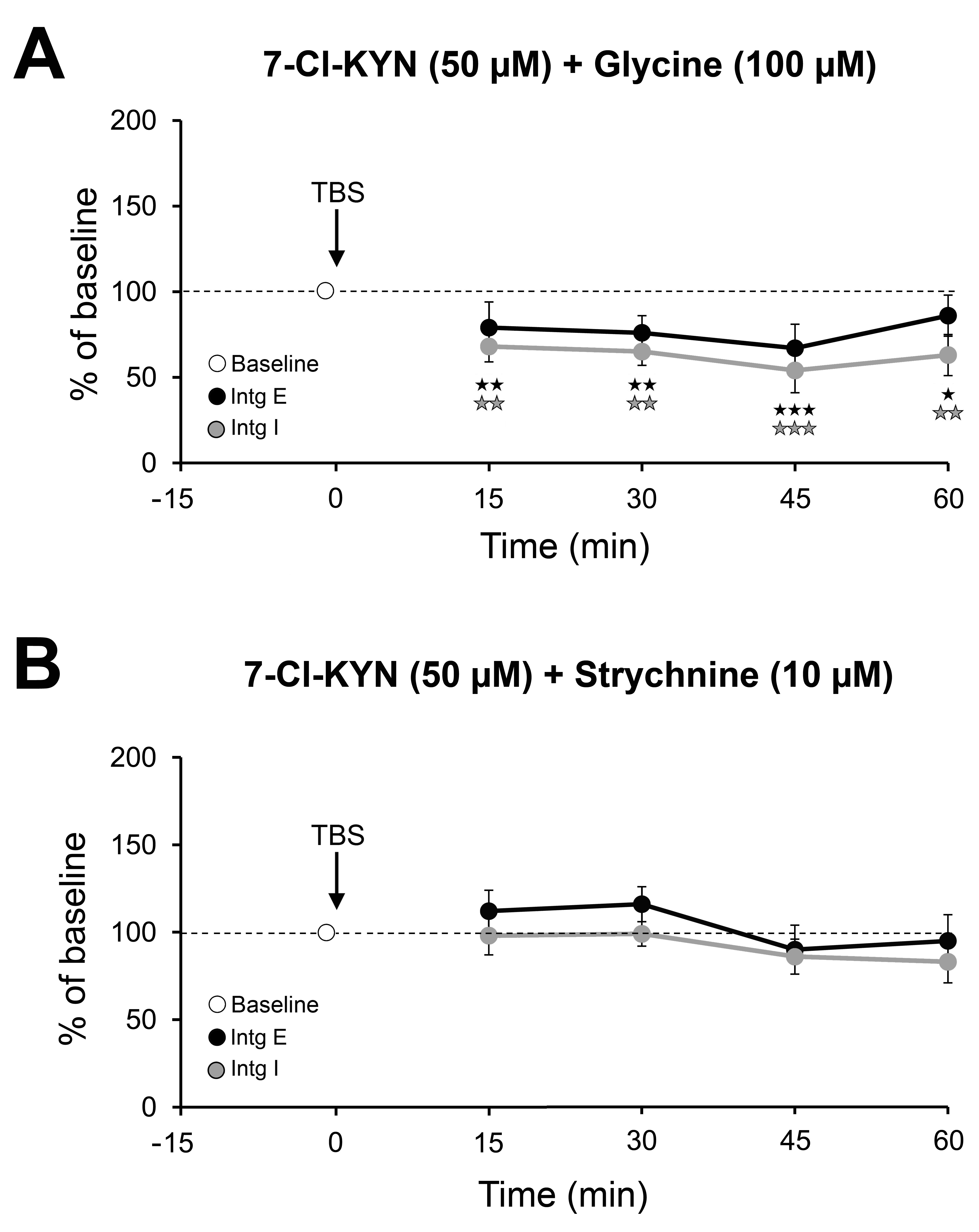

Supplement: S2 Fig — A: The depression observed after TBS administration in the presence of glycine 100μM is not affected by the NMDAR co-agonist binding site blocker 7-Cl-KYN (50μM) indicating that putative regulation of NMDAR by glycine does not play a role in this process (n = 4). B: Instead, GlyRs underlie such downregulation as the GlyRs blocker strychnine (10μM) abolishes the TBS induced depression (n = 4). *p<0.05, **p<0.01, ***p<0.001. (TIF) [file pone.0151233.s002.tif]
